# A novel PCR-based system for the detection of four species of human malaria parasites and *Plasmodium knowlesi*

**DOI:** 10.1371/journal.pone.0191886

**Published:** 2018-01-25

**Authors:** Kanako Komaki-Yasuda, Jeanne Perpétue Vincent, Masami Nakatsu, Yasuyuki Kato, Norio Ohmagari, Shigeyuki Kano

**Affiliations:** 1 Department of Tropical Medicine and Malaria, Research Institute, National Center for Global Health and Medicine, 1-21-1 Toyama, Shinjuku-ku, Tokyo, Japan; 2 Graduate School of Comprehensive Human Sciences, University of Tsukuba, 1-1-1 Tennodai, Tsukuba, Ibaraki, Japan; 3 Disease Control and Prevention Center of National Center for Global Health and Medicine, 1-21-1 Toyama, Shinjuku-ku, Tokyo, Japan; Ehime Daigaku, JAPAN

## Abstract

A microscopy-based diagnosis is the gold standard for the detection and identification of malaria parasites in a patient’s blood. However, the detection of cases involving a low number of parasites and the differentiation of species sometimes requires a skilled microscopist. Although PCR-based diagnostic methods are already known to be very powerful tools, the time required to apply such methods is still much longer in comparison to traditional microscopic observation. Thus, improvements to PCR systems are sought to facilitate the more rapid and accurate detection of human malaria parasites *Plasmodium falciparum*, *P*. *vivax*, *P*. *ovale*, and *P*. *malariae*, as well as *P*. *knowlesi*, which is a simian malaria parasite that is currently widely distributed in Southeast Asia. A nested PCR that targets the small subunit ribosomal RNA genes of malaria parasites was performed using a “fast PCR enzyme”. In the first PCR, universal primers for all parasite species were used. In the second PCR, inner-specific primers, which targeted sequences from *P*. *falciparum*, *P*. *vivax*, *P*. *ovale*, *P*. *malariae*, and *P*. *knowlesi*, were used. The PCR reaction time was reduced with the use of the “fast PCR enzyme”, with only 65 minutes required to perform the first and second PCRs. The specific primers only reacted with the sequences of their targeted parasite species and never cross-reacted with sequences from other species under the defined PCR conditions. The diagnoses of 36 clinical samples that were obtained using this new PCR system were highly consistent with the microscopic diagnoses.

## Introduction

Malaria, the world’s most important life-threating parasitic infectious disease, is caused by protozoan parasites of the genus *Plasmodium* [1]. The species of *Plasmodium* that cause human malaria include *P*. *falciparum*, *P*. *vivax*, *P*. *ovale*, and *P*. *malariae*. In particular, patients with *P*. *falciparum* can rapidly develop high parasitemia, which can have a lethal outcome. The rapid discrimination of *P*. *falciparum* infection from other parasite species is important for providing adequate clinical treatment [2]. *P*. *vivax* and *P*. *ovale* can develop into the dormant hypnozoite stage in the human liver and can cause a relapse of malaria. The hypnozoites that remain in the liver can be eliminated by the administration of primaquine [2]. Thus, the prompt detection and the proper identification of the malaria parasites in a patient’s blood are of the utmost importance for the early and appropriate treatment of malaria. Polymerase chain reaction (PCR)-based methods are recognized as powerful tools for detecting malaria parasites in a patient’s blood [3–6]. In contrast to traditional microscopy-based diagnostic methods, PCR-based methods do not require specialized skills to detect or differentiate parasite species, even in the case of mixed infection [5,6], and they can detect parasites when the parasite density is below the threshold of microscopic detection. One critical disadvantage of using PCR-based methods in the clinical setting is that the time required for PCR-based detection is much longer in comparison to that required for microscopic observation.

A nested PCR targeting the small subunit (SSU) rRNA gene [4], which is in wide use, is the standard method for identifying 4 human malaria species (*Plasmodium falciparum*, *P*. *vivax*, *P*. *ovale*, and *P*. *malariae*). In this method, the first PCR is performed with universal primers designed for the conserved sequence of the small subunit rRNA genes from the *Plasmodium* species. The second PCR is then performed with inner primers designed for the specific sequences of genes from each human malaria parasite species.

Recently, the prevalence of human infection with a simian malaria parasite, *P*. *knowlesi*, has become an important issue in a wide area of Southeast Asia. The identification of this parasite by microscopy is very difficult because it resembles the *P*. *malariae* parasite [7–10]. However, the symptoms caused by *P*. *malariae* and *P*. *knowlesi* are very different, with only *P*. *knowlesi* causing severe and life-threatening malaria.

It has been reported that *P*. *ovale* is clearly divided in two subspecies, *P*. *ovale curtisi* and *P*. *ovale wallikeri*. Although no clinical difference has been found between the infections of these two subspecies, attention should be paid to the diagnosis based on the genetic sequence of these two subspecies [11,12].

Thus, in the present study, the nested PCR method targeting the SSU rRNA genes was improved as follows: 1) the total reaction time of the PCR was reduced to only 65 min using a “fast PCR enzyme”, and 2) the specific primers for the SSU rRNAs of 4 species of human malaria parasites and *P*. *knowlesi* were re-designed.

## Materials and methods

### Patient blood samples

The clinical blood samples used in the present study were obtained from patients who had been diagnosed with suspected malaria at the National Center for Global Health and Medicine (NCGM). The study protocol was carefully reviewed and approved by the NCGM Ethics Committee (No. 1111). The need for participants’ consent was waived by the Ethics Committee.

### Primers

**Fig 1:**
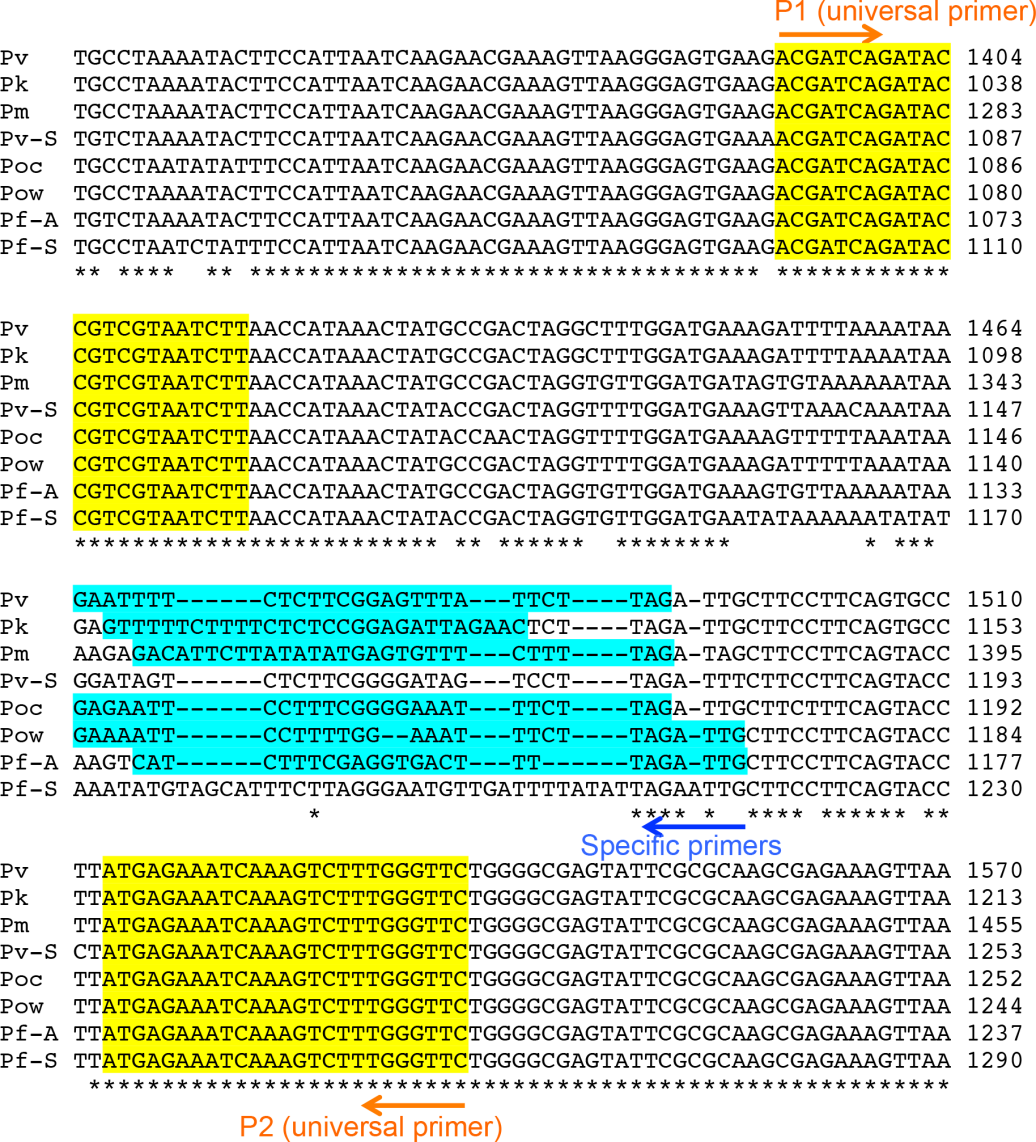
The PCR-targeted region of the SSU rRNA genes from 5 malaria parasite species. Partial sequences of the SSU rRNA genes from malaria parasites are indicated. Pf-S: The sequence from *P*. *falciparum* expressed at the sexual stage [GenBank: M19173]. Pf-A: The sequence from *P*. *falciparum* expressed at the asexual stage [GenBank: M19172]. Pv-S: The sequence from *P*. *vivax* expressed at the sexual stage [GenBank: U03080]. Pv-A: The sequence from *P*. *vivax* expressed at the asexual stage [GenBank: X13926]. Poc: The sequence from *P*. *ovale curtisi* [GenBank: L48986]. Pow: The sequence from *P*. *ovale wallikeri* [GenBank: AB182491] Pm: The sequence from *P*. *malariae* [GenBank: M54897]. Pk: The sequence from *P*. *knowlesi* [GenBank: AY327550]. The universal primers for the conserved region are highlighted in yellow (P1 and P2, see Table 1). The inner-specific primers (F2, V3, M4, Oc4, Ow1, and K1 [see Table 1]) are highlighted in blue.

The sequences of all of the primers used in this study are shown in Table 1 and Fig 1. The universal primers for the SSU rRNA genes of all *Plasmodium* species (P1 and P2) and the specific primers for *P*. *falciparum* (F2) have been previously reported [4]. In this study, the specific primers for the SSU rRNA genes of *P*. *vivax*, *P*. *ovale curtisi*, *P*. *ovale wallikeri*, *P*. *malariae*, and *P*. *knowlesi* were newly designed (V3, Oc4, Ow1, M4, and K1, respectively).

**Table 1:** Sequences of the primers used in the present study.

Type of primer	Primer name	Sequence	Reference
Universal	P1	ACGATCAGATACCGTCGTAATCTT	[4]
Universal	P2	GAACCCAAAGACTTTGATTTCTCAT	[4]
Pf-specific	F2	CAATCTAAAAGTCACCTCGAAAGATG	[4]
Pv-specific	V3	CTAAGAATAAACTCCGAAGAGAAAATTC	This study
Poc-specific	Oc4	CTAAGAAATTTCCCCGAAAGGAATTCTC	This study
Pow-specific	Ow1	CAATCTAAGAAATTTCCAAAAGGAATTTTC	This study
Pm-specific	M4	CTAAAAGAAACACTCATATATAAGAATGTC	This study
Pk-specific	K1	GTTCTAATCTCCGGAGAGAAAAGAAAAAC	This study

Pf, *P*. *falciparum*; Pv, *P*. *vivax*; Poc, *P*. *ovale curtisi*; Pow, *P*. *ovale wallikeri*; Pm, *P*. *malariae*; Pk, *P*. *knowlesi*.

### Plasmids

The plasmids, which contained partial sequences of the SSU rRNA genes of the 5 species of malaria parasites, were constructed as positive control templates for the PCRs. Partial sequences of the SSU rRNA genes from *P*. *falciparum*, *P*. *vivax*, *P*. *ovale curtisi*, and *P*. *malariae* were amplified by a PCR using conventional Taq polymerase and primers P1 and P2. DNA extracted from a cultured parasite laboratory strain, FCR-3 was used for the template of the gene of *P*. *falciparum*. For the amplification of *P*. *vivax*, *P*. *ovale curtisi*, and *P*. *malariae*, parasite DNA samples eluted from clinical patient blood samples were used. The PCR products were then subcloned into plasmid vectors using a TOPO TA Cloning Kit (Life Technologies, Carlsbad, CA, USA). The partial sequence of the SSU rRNA gene from *P*. *ovale wallikeri* and *P*. *knowlesi* was amplified by a PCR using PrimeSTAR GXL DNA polymerase (TaKaRa, Otsu, Japan) with the primers P1 and P2. For the amplification of these standard sequences, parasite DNA samples eluted from clinical patient blood samples were used. Then, the blunt-ended PCR product was subcloned into a plasmid vector using a Zero Blunt TOPO PCR Cloning Kit (Life Technologies). Each subcloned sequence in the plasmid was confirmed by sequencing reactions. The variant sequences of the SSU rRNA gene, which corresponded to the second PCR products, from *P*. *falciparum*, *P*. *vivax*, *P*. *ovale* and *P*. *malariae* were generated by gene synthesis (Eurofins Genomics, Tokyo, Japan). These synthesized genes were supplied as plasmids in which the targeted sequences were inserted in the multi-cloning site of pTAC-2 vector (BioDynamics Laboratory Inc, Tokyo, Japan).

### DNA extraction from patient blood samples

For the PCR with the template DNA from the blood samples of the patients, DNA samples were purified from 200 μL of fresh or frozen blood using a QIAamp DNA Mini Kit (Qiagen, Venlo, Netherlands). We also performed automated DNA extraction using a Maxwell RSC Instrument (Promega, Madison, WI, USA). After the purification step, the DNA samples were eluted with 50 μL or 100 μL of elution buffer.

## The PCR reactions

For the first PCR of the nested PCR, 25 μL of reaction mix containing 1X PrimeSTAR GXL Buffer (TaKaRa), 200 μM each of dNTPs, 0.4 μM each of the P1 and P2 primers, 0.5 μL of PrimeSTAR GXL DNA polymerase (TaKaRa), and 2 μL of the template DNA were mixed. For the positive control, immediately before the experiment, plasmid DNA (which contained the partial sequence of the SSU rRNA gene from each malaria species) was diluted to 0.1 ng/μL (0.2 ng of plasmid DNA per 25 μL of first PCR reaction mix). The conditions of the first PCR were as follows: 35 cycles of 98°C for 10 s, 55°C for 15 s, and 65°C for 5 s. For the second PCR, the products of the first PCR were diluted 1000 times in water. Twenty microliters of the second PCR reaction mix contained 1X PrimeSTAR GXL Buffer, 200 μM each of dNTPs, 0.4 μM of the P1 and each specific inner primer, 0.4 μL of PrimeSTAR GXL DNA polymerase, and 2 μL of diluted first PCR product as a template. The conditions of the second PCR were as follows: 20 cycles of 98°C for 10 s, 58°C for 15 s, and 65°C for 5 s. After the PCR reactions, 5 μL of each PCR product was applied on 2% agarose gels by TAE electrophoresis. GelRed (Wako, Osaka, Japan) was added to the gel prior to electrophoresis for gel staining, and the bands were visualized under UV illumination.

### Determination of the limit of *P*. *falciparum* detection of the nested PCR system

To determinate the limit of detection, a cultured laboratory strain of *P*. *falciparum* was used. The parasite FCR-3 strain was maintained by the method of Trager and Jensen [13]. Parasites were synchronized with 5% D-sorbitol several times [14]. Then, ring-stage parasite-infected erythrocytes were harvested. After the determination of parasitemia by the microscopy, the infected erythrocytes were dissolved in the healthy human whole blood to reach a concentration of 1000 parasites/μL of blood. A series of 10× dilutions was prepared with up to 10^−3^ parasites/μL. The DNA sample was prepared from 200 μL of each blood sample of this dilution series.

## Results and discussion

### The reaction time was reduced with the fast PCR enzyme

The use of the “fast PCR enzyme” in the nested PCR improved its performance. As the expected sizes of the PCR products were small (146–159 bp), the extension time of each PCR cycle could be shortened to only 5 s. The total PCR time was reduced by more than 1 h in comparison with that of the conventional PCR using Taq polymerase [3–5]; the total time needed to perform the first and second PCRs was only 65 min (Table 2). A real-time PCR system can also provide results rapidly [6]; however, our system only requires a PCR system with basic functions.

**Table 2:** Detailed conditions of the nested PCRs.

	PCR with Taq polymerase		PCR with “fast PCR enzyme”	
First PCR	92°C, 2 min			
	92°C, 30 s		98°C, 10 s	
		× 35 cycles	55°C, 15 s	× 35 cycles
	60°C, 90 s		68°C, 5 s	
	60°C, 5 min		4°C	
	4°C			
Time (First PCR)	95 min		40 min	
Second PCR	92°C, 2 min			
	92°C, 60 s		98°C, 10 s	
		× 18 cycles	58°C, 15 s	× 20 cycles
	60°C, 60 s		68°C, 5 s	
	60°C, 5 min		4°C	
	4°C			
Time (Second PCR)	44 min		25 min	
Total Time (First and Second PCR)	139 min		65 min	

## Specificity of the new primer sets for the discrimination of malaria parasite species

**Fig 2:**
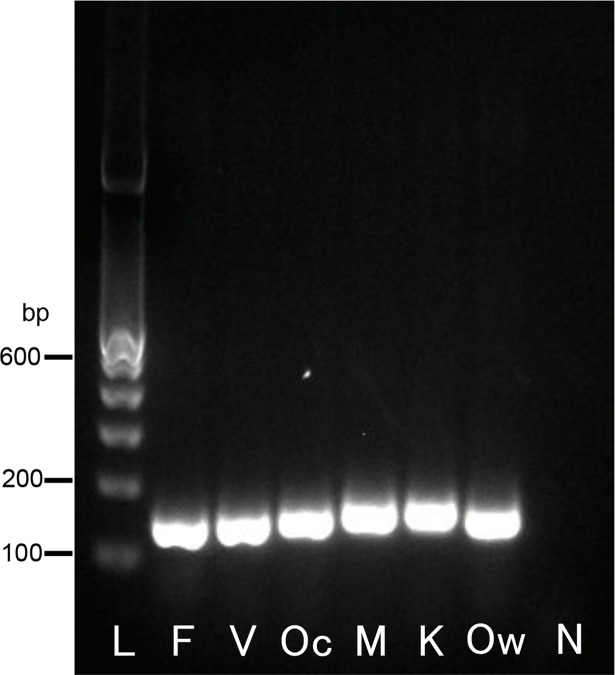
Results of the first PCR with the universal primers P1 and P2. The first PCR products were visualized on 2% agarose-TAE gel containing GelRed (Wako). Lane L indicates a molecular marker (100-bp ladder). The letters shown below each lane indicate the template DNA used for the first PCR reactions (lanes F, V, Oc, M, K, and Ow indicate the plasmid DNA containing each partial sequence of the SSU rRNA genes from *P*. *falciparum*, *P*. *vivax*, *P*. *ovale curtisi*, *P*. *malariae*, *P*. *knowlesi*, and *P*. *ovale wallikeri*, respectively; lane N indicates the negative control [water]). The PCR products are shown at 136–159 bp.

The PCR with universal primers (first PCR) clearly amplified a single band from all types of template DNA (Fig 2). A gradient PCR machine was not needed for the second PCR, as the conditions (including the annealing temperatures) were the same for all of the primer sets.

**Fig 3:**
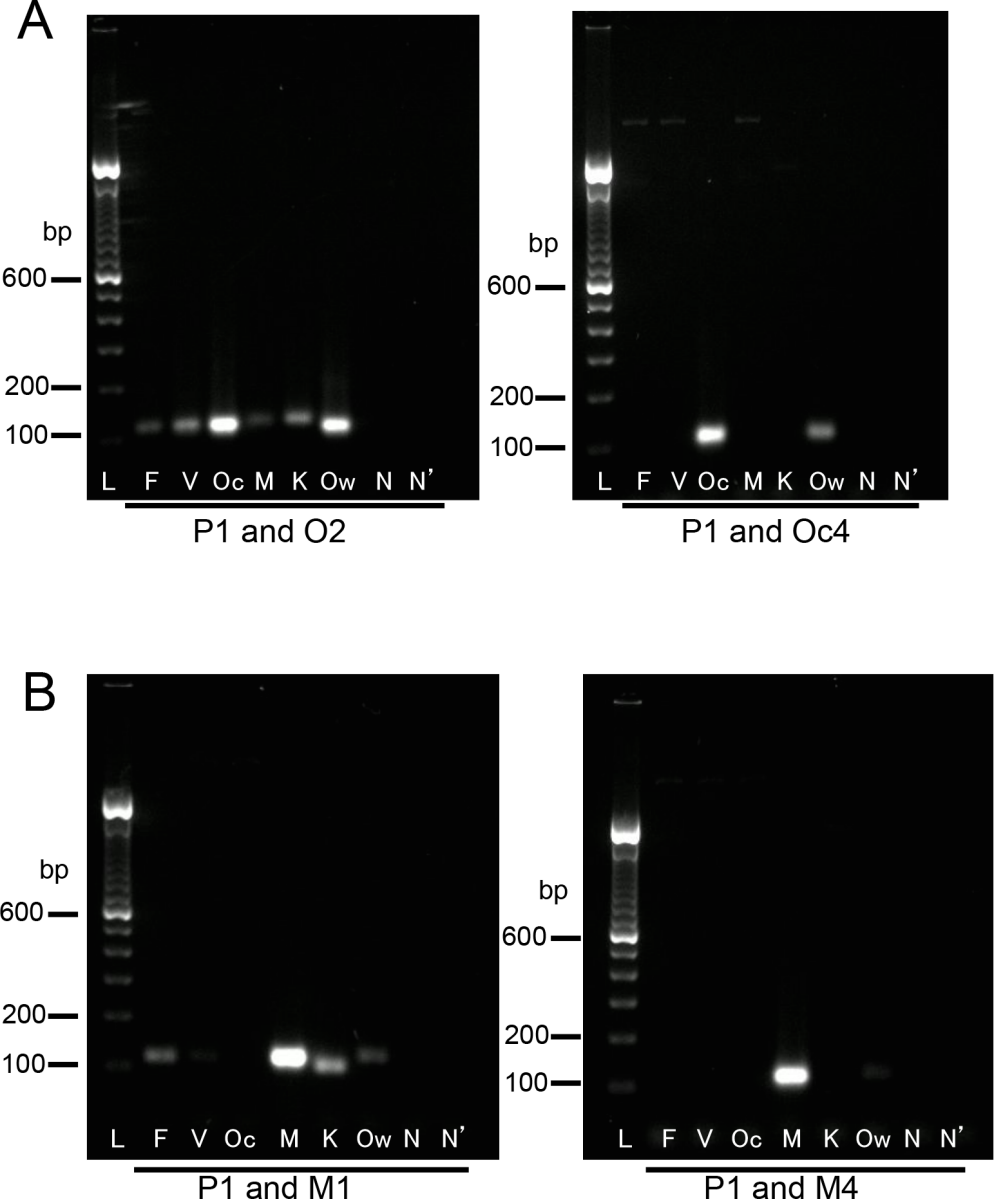
Comparison of the performance of the classic and new primer sets in the second PCR. (**A**) Comparison of the classic O2 primer and new Oc4 primer. (**B**) Comparison of the classic M1 primer and new M4 primer. The second PCR was performed with 30 cycles. The PCR products were visualized on 2% agarose-TAE gel containing GelRed (Wako). Lane L indicates a molecular marker (100-bp ladder). The letters shown below each lane indicate the template used for the second PCR reactions (lanes F, V, Oc, M, K, and Ow indicate diluted first PCR products amplified from the sequences of *P*. *falciparum*, *P*. *vivax*, *P*. *ovale curtisi*, *P*. *malariae*, *P*. *knowlesi*, and *P*. *ovale wallikeri*, respectively; lane N indicates the diluted first PCR product from water; lane N’, water). The primer sets are indicated below the gels.

With the PCR enzyme used in this study, the *P*. *ovale-* and *P*. *malariae*-specific primers used in the previous study [4] sometimes cross-reacted with the sequences from other species. No cross-reaction occurred with these primers when the conventional Taq polymerase was used for the reaction (data not shown); thus, the powerful proof-reading activity of the PCR enzyme may cause such cross-reactions. In addition, the previously reported *P*. *ovale-*specific primer was designed based on the sequence of *P*. *ovale curtisi*, one of the subspecies of *P*. *ovale*. Thus, new primers specific to the SSU rRNA genes from two subspecies of *P*. *ovale* (*P*. *ovale curtisi* and *P*. *ovale wallikeri*) and *P*. *malariae* were designed (primers Oc4, Ow1, and M4, respectively: Table 1). The comparison of the sets of the classic and new primers specific for *P*. *ovale curtisi* and *P*. *malariae* clearly showed the relatively high sensitivity of the new primers in the performance of the second PCR reactions with the increase to 30 cycles. With the primer set of P1 and the classic O2, PCR products were amplified from the sequence of all species. With the primer set of P1 and the new Oc4, PCR products were amplified solely from the sequences of *P*. *ovale curtisi* and *P*. *ovale wallikeri* (Fig 3A). With the primer set of P1 and the classic M1, PCR amplifications were detected from the specific sequences of *P*. *falciparum*, *P*. *malariae*, *P*. *knowlesi*, and *P*. *ovale wallikeri*. However, with the primer set of P1 and the new M4, PCR products were amplified only from the specific sequence of *P*. *malariae* (Fig 3B).

The previously reported *P*. *vivax*-specific primer was reported to cross-react with the SSU rRNA gene from *P*. *knowlesi* [10]. Thus, specific primers for the SSU rRNA genes from *P*. *vivax* and *P*. *knowlesi* were newly designed in the present study (primers V3 and K1; Table 1).

To verify the specificity of the newly designed nested PCR system, a PCR with the universal P1 primer and each of the specific F2, V3, Oc4, Ow1, M4, and K1 primers was performed with the first PCR products, which were amplified from each of the SSU rRNA genes from every species. In the second PCR, the combinations of the universal primer (P1) and each of the inner-specific primers for *P*. *falciparum*, *P*. *vivax*, *P*. *ovale curtisi*, *P*. *malariae*, *P*. *knowlesi*, and *P*. *ovale wallikeri* (F2, V3, Oc4, M4, and K1, respectively) clearly resulted in the amplification of a single band from the first PCR products, corresponding to each positive control, and never cross-reacted with the first PCR products amplified from the partial sequences of the other species of *Plasmodium*.

**Fig 4:**
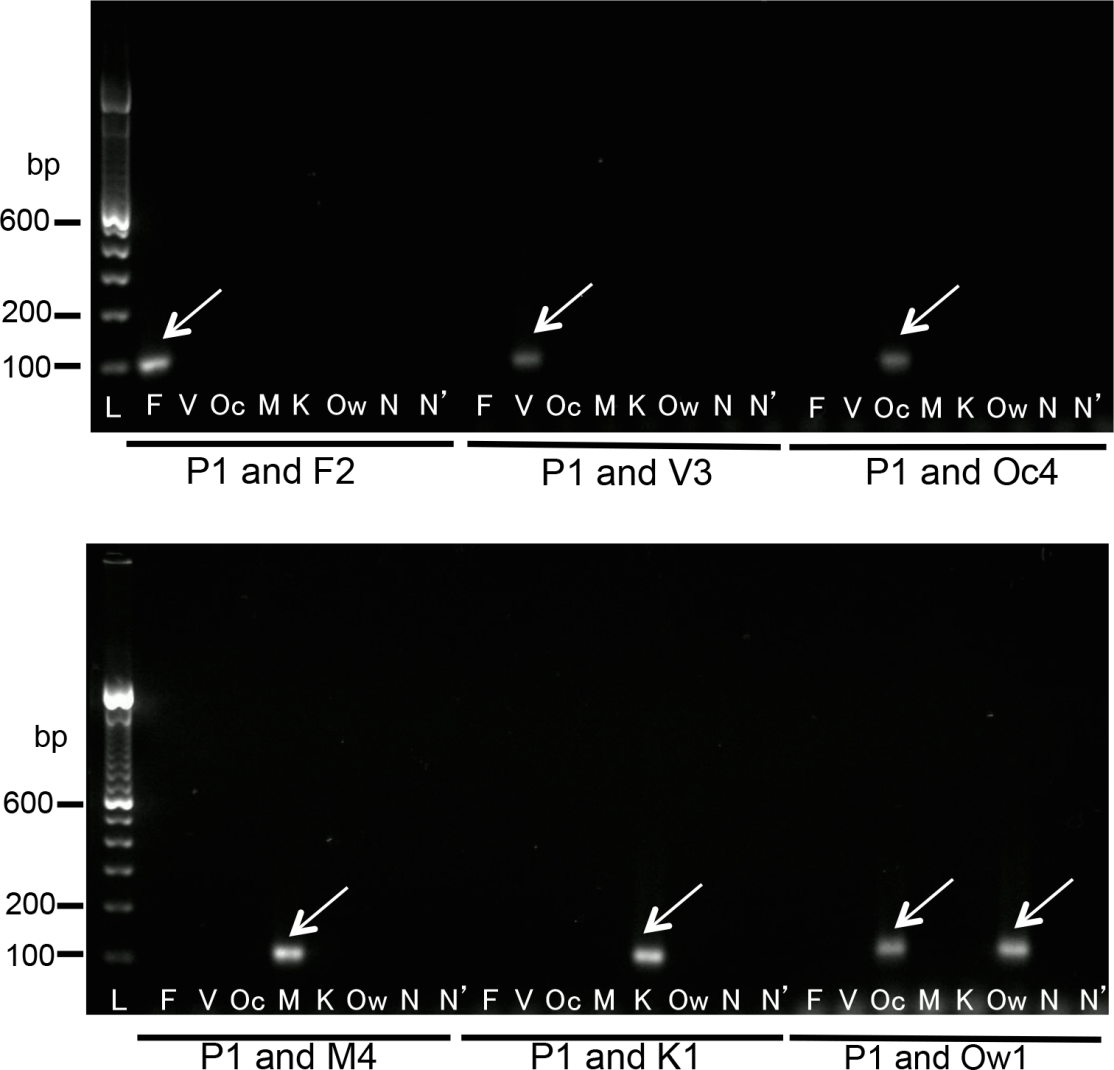
Results of the second PCR with the universal primer P1 and the inner-specific primers. The second PCR products were visualized on 2% agarose-TAE gel containing GelRed (Wako). Lane L indicates a molecular marker (100-bp ladder). The letters shown below each lane indicate the template used for the second PCR reactions (lanes F, V, Oc, M, K, and Ow indicate diluted first PCR products amplified from the sequences of *P*. *falciparum*, *P*. *vivax*, *P*. *ovale curtisi*, *P*. *malariae*, *P*. *knowlesi*, and *P*. *ovale wallikeri*, respectively; lane N indicates the diluted first PCR product from water; lane N’, water). Arrows indicate the PCR products (100–106 bp). The primer sets are indicated below the gels.

An exceptional case was observed with the specific primer for *P*. *ovale wallikeri*. This Ow1 primer clearly amplified the sequence *P*. *ovale wallikeri* and cross-reacted of that of *P*. *ovale curtisi* (Fig 4). Thus, this system cannot discriminate between the *P*. *ovale curtisi* single infection and the mixed infection of the two subspecies, *P*. *ovale curtisi* and *P*. *ovale wallikeri*. Cases that were positive with both the Oc4 and Ow1 primers in the second PCR were simply classified as “*P*. *ovale* infection”. No clinical problem is expected from the difficulty of differentiation of these two subspecies. For more detailed discrimination of the infection of these two subspecies, sequencing analysis of the PCR products would be required.

**Fig 5:**
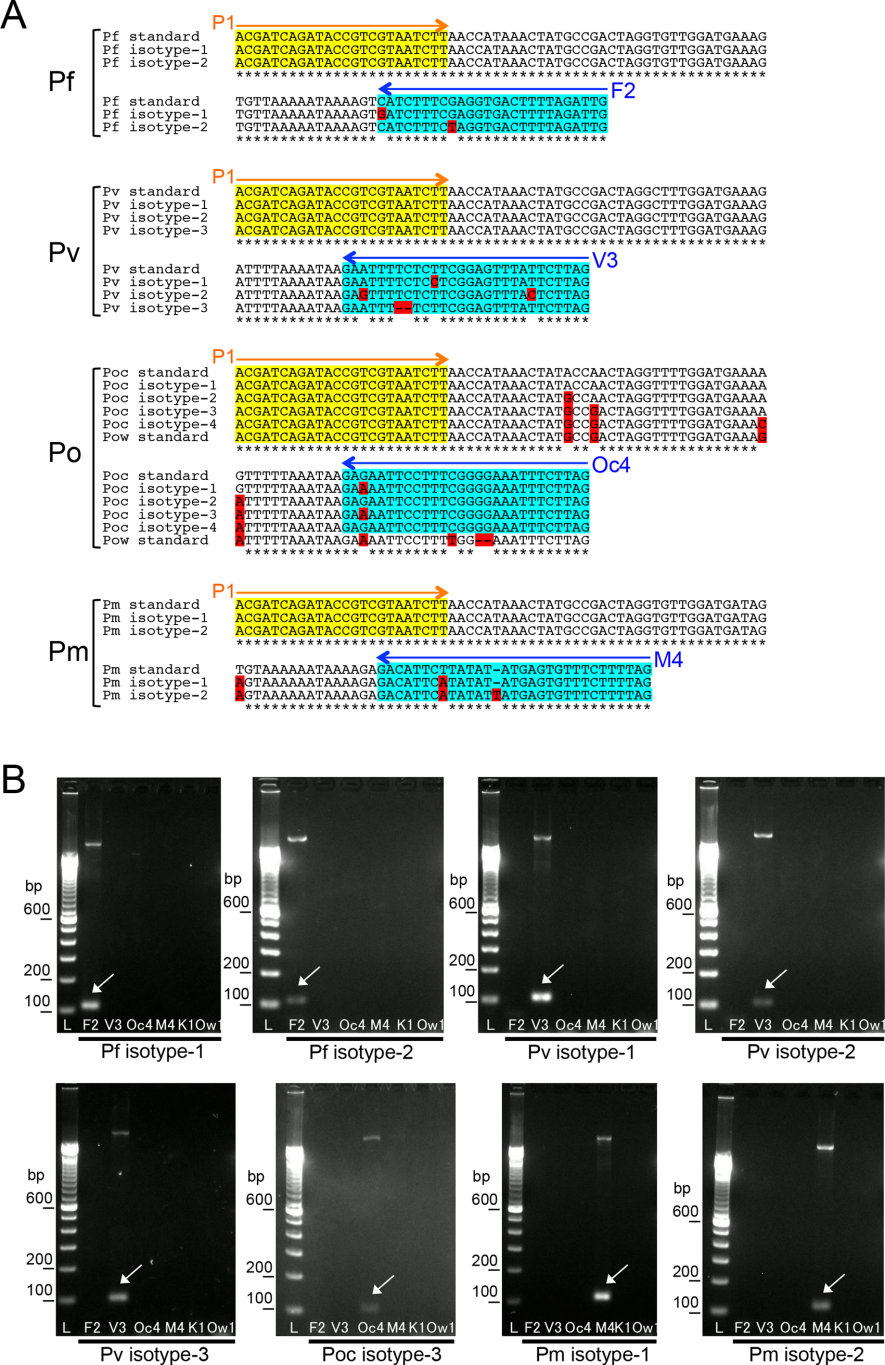
The second PCR-targeted region of the SSU rRNA genes from variant parasite isotypes. **(A)** Partial sequences of the variant SSU rRNA genes from malaria parasites. Pf, Pv, Po, and Pm indicate *P*. *falciparum*, *P*. *vivax*, *P*. *ovale* and *P*. *malariae* respectively. Pf-standard: The sequence from *P*. *falciparum* [GenBank: M19172]. Pf isotype-1 and isotype-2: The identified variant sequences from *P*. *falciparum* [Genbank: KJ170099.1 and JQ627151.1]. Pv-standard: The sequence from *P*. *vivax* [GenBank: X13926]. Pv isotype-1, isotype-2 and isotype-3: The identified variant sequences from *P*. *vivax* [Genbank: U83877.1, KC750244.1 and AF145335.1]. Poc standard: The sequence from *P*. *ovale curtisi* [GenBank: L48986]. Poc isotype-1, isotype-2, isotype-3 and isotype-4: The identified variant sequences from *P*. *ovale curtisi* [Genbank: KF696376.1, KC633228.1, KJ871671.1 and KF696371.1]. Pow standard: The sequence from *P*. *ovale wallikeri* [GenBank: AB182491] Pm standard: The sequence from *P*. *malariae* [GenBank: M54897]. Pm isotype-1 and isotype-2: The identified variant sequences from *P*. *malariae* [Genbank: KJ619947.1 and KJ170106.1]. All variant sequences from each species are indicated as multiple alignment comparisons. The universal P1 primer region is highlighted in yellow (P1). The inner-specific primers (F2, V3, Oc4 and M4,) are highlighted in blue. The nucleotide changes identified in each variant are highlighted in red. **(B)** Results of the PCR with the universal primer P1 and the inner-specific primers using the variant DNAs as templates. The PCR reactions were performed with the universal P1 primer and each species-specific primer. The PCR conditions were same as those of the second PCR (see Materials and Methods). In each reaction mix, 0.1 ng of the synthesized DNA of each variant sequence was included as template. The products were visualized on 2% agarose-TAE gel containing GelRed (Wako). Lane L indicates a molecular marker (100-bp ladder). The letters shown below each lane indicate the specific primer used for the second PCR reactions (see Table 1). Arrows indicate the PCR products (100–106 bp). The template DNAs are indicated below the gels.

In addition, variations of SSU rRNA gene sequence among multi-copies in the genome of same species or genetic isotypes of a *Plasmodium* species had been deposited in gene databases. To investigate the second PCR reactivity for each variant DNA, the NCBI nucleotide collection database (https://www.ncbi.nlm.nih.gov/nucleotide) was searched using the representative sequences of the second PCR products from each species. As a result, several patterns of variant for *P*. *falciparum* (2 isotypes), *P*. *vivax* (3 isotypes), *P*. *ovale curtisi* (4 isotypes) and *P*. *malariae* (2 isotypes) were additionally identified (Fig 5A). No additional variants of the region were detected for *P*. *ovale wallikeri* or *P*. *knowlesi*. Some of these variants contain one or two nucleotide changes inside the region covered by the specific primer. The DNA sequences of these additional variants were generated by artificial gene synthesis. For the *P*. *ovale curtisi* variants, the two isotypes did not have nucleotide changes inside the region of the Oc4 primer (Poc isotype-2 and Poc isotype-4; Fig 5A); the others had the same single nucleotide change inside this region (Poc isotype-1 and Poc isotype-3; Fig 5A). Then, from *P*. *ovale curtisi* variants, only the sequence of Poc isotype-3 was generated to investigate the reactivity of the Oc4 primer to this region. The PCR with the species-specific primers was performed using these synthesized DNAs as templates. As a result, in spite of the one or two base-pair changes inside the primer region, all of the template DNAs were amplified solely by the primer targeted for the specific species (Fig 5B). These results indicated that the potential application of the nested-PCR system in the discrimination of *Plasmodium* species in unknown clinical samples.

In addition to *P*.*knowlesi*, the possibility of the outbreak of simian malaria parasites in humans has become a concern [15]. For example, a case in which a human was naturally infected with *P*. *cynomolgi* was reported in 2014 [16]. *P*. *cynomolgi* is closely genetically related to *P*. *vivax* and morphologically indistinguishable from *P*. *vivax* under microscopic observation [16]. From this point of view, in the region corresponding to the *P*. *vivax* specific V3 primer, the SSU rRNA gene sequences from *P*. *cynomolgi* isolates have only three nucleotides differences from the sequence of the V3 primer (S1 Fig). Thus, it is expected to be difficult to clearly discriminate *P*. *cynomolgi* from *P*. *vivax* using our system. To diagnose such rare zoonotic malaria cases, it is important to pay careful attention to the patient’s symptoms, travel history and the environmental characteristics of the area where he/she stayed, and if necessary, to perform sequencing of the longer region of the SSU rRNA gene.

### Detection limit for *P*. *falciparum*-infected erythrocytes in the nested PCR system

**Fig 6:**
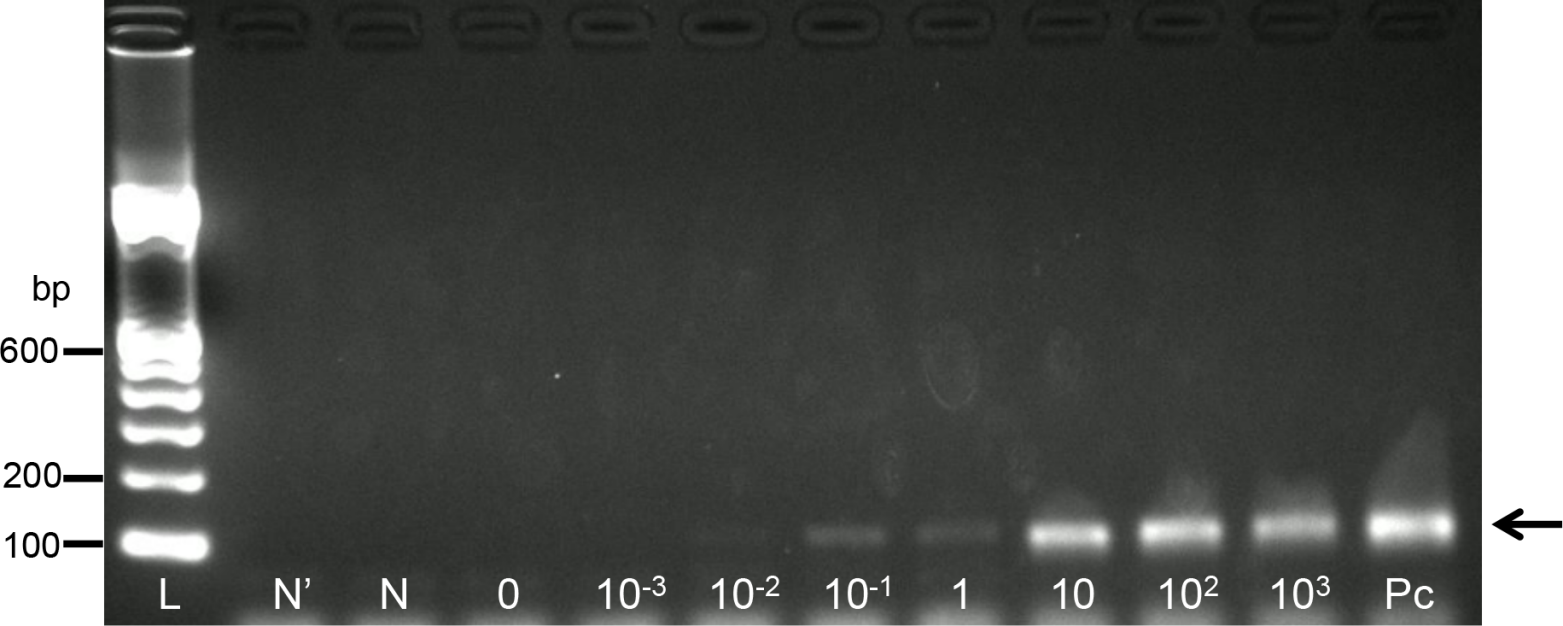
Determination of the *P*. *falciparum* detection limit of the nested PCR system. The second PCR products were visualized on 2% agarose-TAE gel containing GelRed (Wako). The primer set used for the second PCR was P1 and F2. Lane L indicates a molecular marker (100-bp ladder). The letters shown below each lane indicate the template used for the second PCR reactions (lane P indicates the first PCR products amplified from the sequences of *P*. *falciparum*; lanes 10^3^, 10^2^, 1, 10^−1^, 10^−2^, and 10^−3^ indicate the first PCR products amplified from the DNA extracted from the blood sample containing 10^3^, 10^2^, 1, 10^−1^, 10^−2^, and 10^−3^ parasites/μL of blood, respectively; lane 0 indicates the first PCR products amplified from the DNA extracted from healthy blood; lane N indicates the diluted first PCR product from water; lane N’, water). The *P*. *falciparum-*specific PCR products are indicated with an arrow on the right side.

The detection limit of the nested PCR system was determined using cultured *P*. *falciparum*. The series of diluted samples of ring-stage parasites were used for the template DNA extraction. In the second PCR with the *P*. *falciparum*-specific primer, a clear band was observed until the DNA was extracted from the blood sample with 0.1 parasite/μL (Fig 6). This parasite concentration is approximately equivalent to 2 × 10^−6^% parasitemia. Thus, for *P*. *falciparum*, this nested PCR system could detect parasites in samples with submicroscopic parasitemia.

## Parasite determination in the clinical blood samples

**Fig 7:**
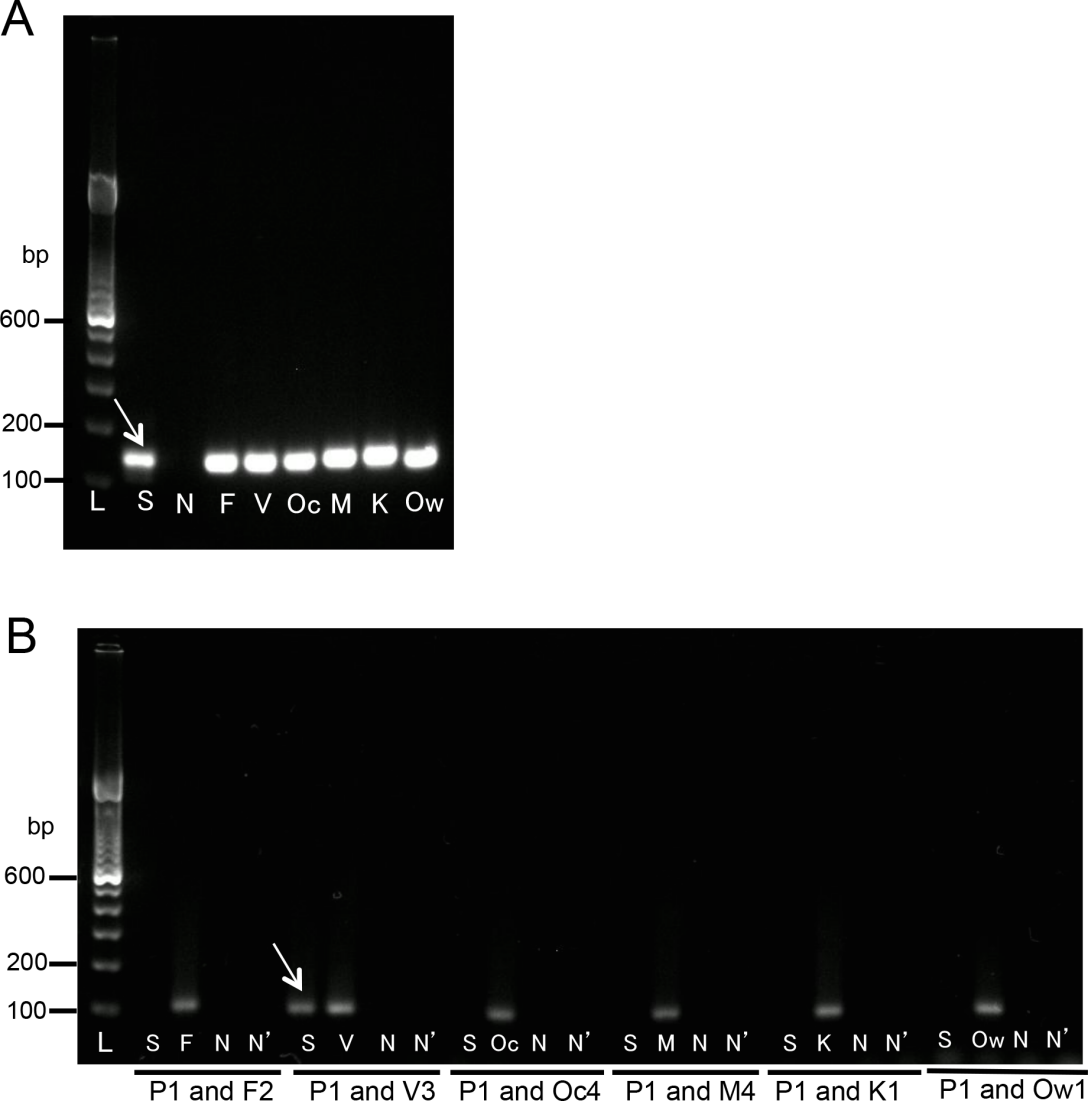
Gel electrophoresis of PCR products for the diagnosis of a clinical sample. (**A**) The first PCR products were visualized on 2% agarose-TAE gel containing GelRed (Wako). Lane L indicates a molecular marker (100-bp ladder). The letters shown below each lane indicate the template DNA used for the first PCR reactions (lane S indicates a DNA sample from a patient’s blood; lanes F, V, Oc, M, K, and Ow indicate plasmid DNA containing each partial sequence of the SSU rRNA genes from *P*. *falciparum*, *P*. *vivax*, *P*. *ovale curtisi*, *P*. *malariae*, *P*. *knowlesi*, and *P*. *ovale wallikeri*, respectively; lane N, indicates the negative control [water]). The PCR products are shown at 136–159 bp. (**B**) The second PCR products were visualized on 2% agarose-TAE gel containing GelRed (Wako). Lane L indicates a molecular marker (100-bp ladder). The letters shown below each lane indicate the template used for the second PCR reactions (lane S indicates the diluted first PCR product amplified from a DNA sample from a patient’s blood; lanes F, V, Oc, M, K, and Ow indicate the diluted first PCR products amplified from the sequences of *P*. *falciparum*, *P*. *vivax*, *P*. *ovale curtisi*, *P*. *malariae*, *P*. *knowlesi*, and *P*. *ovale wallikeri*, respectively; lane N indicates the diluted first PCR product from water; lane N’, water). The arrow indicates the PCR product amplified from a DNA sample from the blood of a patient with *P*. *vivax*-specific primers. The primer sets are indicated below the gel.

Finally, the species of the malaria parasites in the patients’ clinical blood samples were discriminated using the new nested PCR system, and the results were compared with those obtained by microscopic observation (Table 3). The diagnoses of the 36 cases that were obtained with the new PCR showed good consistency with the microscopy-based diagnoses. Of the 31 cases that were already diagnosed as being malaria positive by microscopic observation, 31 cases were also diagnosed as malaria positive by the new nested PCR system. The parasite species detected by the specific secondary PCR was completely consistent with the species identified by microscopic observation. A representative example of the gel electrophoresis results is shown in Fig 7 (Case No. 24, Table 3). The 31 PCR-positive cases included *P*. *falciparum* (n = 23), *P*. *vivax* (n = 2), *P*. *ovale* (n = 3), *P*. *ovale wallikeri* (n = 1), *P*. *malariae* (n = 1), and *P*. *knowlesi* (n = 1). Five cases that were microscopy negative were also PCR negative.

**Table 3:** Detection of the 5 species of human malaria parasite in the patient blood samples.

	Species detected by			
Case No.	Microscopic observation (% parasitemia)	Nested PCR	Method of DNA Extraction ^a^	Country of origin
1	Pf (0.36%)	Pf	A'	Malawi
2	Pf (0.5%)	Pf	B'	Sierra Leone
3	Pf (16.52%)	Pf	B'	Guinea
4	Pf (0.05%)	Pf	B'	Liberia
5	Pf (0.22%)	Pf	B'	Congo
6	Pf (0.44%)	Pf	B'	Cameroon
7	Pf (0.725%)	Pf	B'	Uganda
8	Pf (0.023%)	Pf	B'	Uganda
9	Pf (0.21%)	Pf	B'	Papua New Guinea
10	Pf (1.78%)	Pf	B'	Côte d'Ivoire
11	Pf (0.085%)	Pf	B'	Chad
12	Pf (0.1%)	Pf	B'	Mozambique
13	Pf (0.055%)	Pf	B'	Ghana
14	Pf (0.018%)	Pf	B'	Ghana
15	Pf (0.0094%)	Pf	B'	Cameroon
16	Pf (0.34%)	Pf	B'	Zambia
17	Pf (0.068%)	Pf	B'	Congo
18	Pf (1.052%)	Pf	B'	Ivory Coast
19	Pf (0.011%)	Pf	B	Cameroon
20	Pf (4.5%)	Pf	B	Ivory Coast
21	Pf (2.1%)	Pf	B	Ivory Coast
22	Pf (1.8%)	Pf	B	Angola
23	Pf (0.15%)	Pf	B	Angola
24	Pv (0.24%)	Pv	A	Papua New Guinea
25	Pv (0.16%)	Pv	A	India
26	Po (0.07%)	Po ^d^	A	Cameroon
27	Po (0.028%)	Po ^d^	A	Zambia
28	Po (0.038%)	Po ^d^	A	Ghana
29	Po (0.0015%)	Pow	B	Angola
30	Pm (0.13%)	Pm	A	Ivory Coast
31^b^	Pm or Pk (0.2%)	Pk	A	Malaysia
32	Negative	Negative	A	ND
33	Negative	Negative	A	India
34^c^	Negative	Negative	B	Angola
35	Negative	Negative	B	Philippines
36	Negative	Negative	B	Philippines

Pf, *P*. *falciparum*; Pv, *P*. *vivax*; Po, *P*. *ovale*; Pm, *P*. *malariae*; Pk, *P*. *knowlesi*, Pow; *P*. *ovale wallikeri*.

^a^ Each DNA extraction method is shown. A, DNA was eluted from fresh blood using a DNA mini-kit (Qiagen); A’, DNA was eluted from frozen blood using a DNA mini-kit; B, DNA was eluted from fresh blood using a Maxwell RSC Instrument (Promega); B’, DNA was eluted from frozen blood using a Maxwell RSC Instrument.

^b^ In case no. 31, *P*. *knowlesi* infection was already verified in a previous report using a PCR targeting the cytochrome *b* gene [10].

^c^ The ICT test showed that case no. 34 was positive for *P*. *falciparum*.

^d^ The three *P*. *ovale-*positive cases appeared in the results of the second PCR, which were positive with the primer sets for both *P*. *ovale curtisi* and *P*. *ovale wallikeri*.

Case no. 34 (Table 3) was found to be negative by the nested PCR system, despite the fact that *P*. *falciparum* was detected in the same blood sample by the NOW Malaria rapid-immunochromatographic test (ICT) (Binax, Inc., Portland, ME, USA). The *P*. *falciparum*-specific band (T1 band) of the ICT test was positive against the HRP-2 antigen. It is known that the T1 band sometimes remains positive after treatment, even in cases in which the parasite is not detected by microscopy, because HRP-2 sometimes persists in the bloodstream after the clearance of the parasite [17]. In case no. 34, we hypothesized that a previous *P*. *falciparum* infection might have caused a false-positive result in the ICT test. Taken together, we may conclude that a nested PCR-based diagnosis is superior for the diagnosis of true-negative cases.

## Conclusion

To date, various methods have been developed to diagnose malaria. Among these, the nested PCR has been considered to be the definitive method for discriminating malaria parasite species in the human blood stream. Our results indicated that the nested PCR method with new primer sets showed improved sensitivity and specificity. Moreover, in the improved nested PCR, only 65 min are required for the first and second reactions. This new system could become a standard nested PCR that can be used to accurately diagnose clinical samples.

## Supporting information

S1 Fig The second PCR-targeted region of the SSU rRNA genes from *P*. *vivax* and *P*. *cynomolgi* isotypes. All sequences are indicated as multiple alignment comparisons. Pv-standard: The sequence from *P*. *vivax* [GenBank: X13926]. Pv isotype-1, isotype-2 and isotype-3: The identified variant sequences from *P*. *vivax* [Genbank: U83877.1, KC750244.1 and AF145335.1]. Pc isotype-1 and isotype-2: The variant sequences from *P*. *cynomolgi* [Genbank: L07559.1 and AB287290.1]. The universal P1 primer region is highlighted in yellow (P1). The inner *P*. *vivax*-specific primer (V3) is highlighted in blue. The nucleotide changes identified in each variant were highlighted in red.
(TIF)
