# Supplementary figures and images for: A novel PCR-based system for the detection of four species of human malaria parasites and Plasmodium knowlesi
Source: PLoS One. 2018 Jan 25;13(1):e0191886. doi: 10.1371/journal.pone.0191886 (PMC5785027; doi:10.1371/journal.pone.0191886)

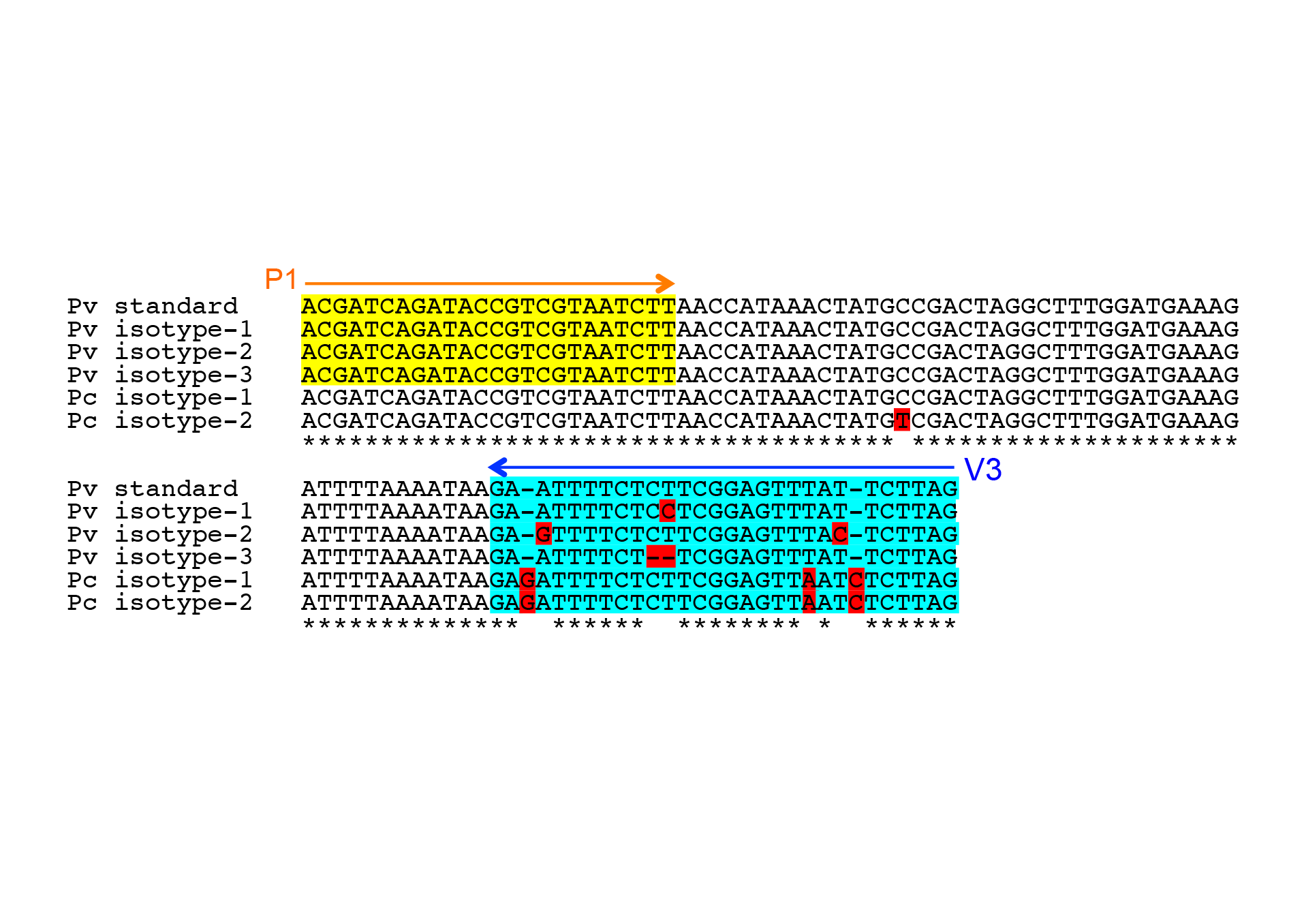

Supplement: S1 Fig — All sequences are indicated as multiple alignment comparisons. Pv-standard: The sequence from P. vivax [GenBank: X13926]. Pv isotype-1, isotype-2 and isotype-3: The identified variant sequences from P. vivax [Genbank: U83877.1, KC750244.1 and AF145335.1]. Pc isotype-1 and isotype-2: The variant sequences from P. cynomolgi [Genbank: L07559.1 and AB287290.1]. The universal P1 primer region is highlighted in yellow (P1). The inner P. vivax-specific primer (V3) is highlighted in blue. The nucleotide changes identified in each variant were highlighted in red. (TIF) [file pone.0191886.s001.tif]
